# Supplementary material for: WordCommentsAnalyzer: A windows software tool for qualitative research
Source: F1000Res. 2018 Sep 4;7:536. Originally published 2018 May 3. [Version 2] doi: 10.12688/f1000research.14819.2 (PMC6137411; doi:10.12688/f1000research.14819.2)
Supplement: Supplementary file 3 [file f1000research-7-17614-s0002.tgz › b2a79496-4ada-47c4-9682-a99ce867836d.zip!/Eight Iranian Health Journals/The Journal of Toloo-e-Behdasht_16.docx]

© The Journal of Toloo-e-Behdasht. This work is licensed under CC BY 4.0 (https://creativecommons.org/licenses/by/4.0/).

Volume & Issue: دوره 14 - شماره 5

Title: بررسی وضعیت سلامت و نیازهای بهداشتی سالمندان استان ایلام در سال 1391

http://tbj.ssu.ac.ir/article-1-1899-fa.html

مقدمه: دوره سالمندی نیازها و مشکلات بهداشتی مخصوص به خود دارد. پژوهش حاضر با هدف تعیین مسائل و نیازهای بهداشتی سالمندان استان ایلام در سال 1391 انجام شده است. روش‌‌بررسی: در این مطالعه توصیفی-تحلیلی، 1012 سالمند ساکن در شهرستان‌های هشت‌گانه استان ایلام بر‌اساس تخصیص متناسب، یک پرسشنامه‏‌ استاندارد را از طریق مصاحبه چهره به چهره تکمیل کردند. داده های گردآوری‌شده با استفاده از نرم‏افزار SPSS مورد تجزیه و تحلیل قرار گرفت. یافته‌ها: از مجموع 1012 سالمند مورد مطالعه (5/60٪) مرد و (5/39٪) زن بودند. میانگین سنی شرکت‌کنندگان 02/8±1/71 سال و بیشتر سالمندان مورد مطالعه در گروه سنی 69-65 سال بودند. 6/38% تحت پوشش سازمان بیمه خدمات درمانی بوده اند. میانگین فشار خون سیستولیک و دیاستولیک افراد مورد مطالعه به‌‌ترتیب 38/13 و 76/11 بود. میانگین توده بدنی افراد مورد مطالعه 72/25 بود. 1/36% سابقه ابتلا به بیماری‌های قلبی، 15 % دیابت، 5/22% مشکل تنفسی و 3/16% به سیگار اعتیاد داشتند. 3/45% سابقه جراحی، 8/72% سابقه درد مفاصل، 6/64% سابقه درد اسکلتی و 6/12% سابقه سکته مغزی داشتند. افتادن از ارتفاع بیشترین (18%) فراوانی سوانح و حوادث را به خود اختصاص داده بود. نتیجه‌گیری: مشکلات شایع جسمی سالمندان استان ایلام عمدتا" به بیماریهای غیرواگیر و بویژه قلبی-عروقی و دیابت بر می‌گردد. بنا‌براین لزوم اجرای مداخلات  ارتقا سلامت برای سالمندان به منظور داشتن یک زندگی سالمندی سالم ضروری به نظر می‌رسد.

Title: بررسی کارایی روش توام انعقاد شیمیایی و اکسیداسیون پیشرفته (پروکسون) در تصفیه فاضلاب رنگی کارخانجات لوازم خانگی

http://tbj.ssu.ac.ir/article-1-1898-fa.html

چکیده مقدمه: نظر به نقش مهم صنایع در آلوده‌سازی محیط، پژوهش حاضر به منظور بررسی کارایی روش تلفیقی انعقاد شیمیایی و پروکسون (O3/H2O2) در تصفیه فاضلاب رنگی کارخانجات لوازم خانگی طراحی شده است. روش‌بررسی: این پژوهش از نوع تجربی بوده و در مقیاس آزمایشگاهی بر روی فاضلاب واحد رنگ کاری کارخانه تولید لوازم خانگی انجام پذیرفت. نمونه برداری به شیوه مرکب انجام شد. فرآیندهای مورد استفاده فرآیند انعقاد شیمیایی و اکسیداسیون پیشرفته (پروکسون) می‌باشد. در این تحقیق تعداد 250 نمونه مورد آزمایش قرار گرفت. جهت انجام مرحله انعقاد شیمیایی از منیزیم کلراید، پلی آلومینیوم کلراید و آهن III کلرید، پلیمر کاتیونی و بنتونیت استفاده شد. در این تحقیق از دستگاه هایی نظیر اسپکتروفتومتر مدل DR 2000، جارتست و رآکتور ازن زنی استفاده گردید و بر روی نمونه‌ها آزمایشاتی نظیر COD و رنگ سنجی مطابق دستورالعمل Standard Method بر روی نمونه‌ها صورت پذیرفت. یافته‌ها: نتایج مطالعه حاضر نشان داد که هر یک از منعقد‌کننده‌های مورد بررسی در pH بهینه خود توانستند به ترتیب 85/86 % (منیزیم‌کلراید)، 47/88% (پلی‌آلومینیوم‌کلراید) و 41/85% (آهن III کلرید) در حذف COD تاثیر‌گذار باشند. پلی‌آلومینیوم کلراید با 92/90% بالاترین راندمان حذف رنگ را حاصل نمود. همچنین بالاترین راندمان حذف COD مربوط به ترکیب منیزیم کلراید (mg/l 4/1)، پلی آلومینیوم کلراید (mg/l 6/0) و پلیمر‌های کاتیونی (mg/l 4/0) با راندمان 11/89% بود که رنگ را نیز به میزان 38/93% حذف نمود. با انجام فرآیند اکسیداسیون پیشرفته به روش پروکسون بر روی فاضلاب پیش تصفیه شده در مرحله انعقاد شیمیایی راندمان حذف COD از 11/89% به 67/99% رسید. نتیجه‌گیری: به دلیل حالت سوسپانسیونی این نوع فاضلاب می‌بایستی در ابتدا جامدات محلول موجود در فاضلاب حذف شده و سپس فرآیند‌های تکمیلی به منظور تصفیه بهتر انجام پذیرد. از این رو فرآیند پروکسون در ترکیب با فرآیند انعقاد شیمیایی دارای قابلیت بسیار بالایی در حذف COD و رنگ از فاضلاب کارخانجات لوازم خانگی می‌باشد.

Title: متا‌آنالیز تأثیر مکمل یاری ویتامین D بر مقاومت به انسولین در مبتلایان به دیابت

http://tbj.ssu.ac.ir/article-1-1900-fa.html

مقدمه: دیابت نوع 2 و کمبود ویتامین D هر دو از بیماری‌های شایع هستند. پژوهش‌های پرشمار ارتباط میان این دو را بررسی کرده اند. هدف این مطالعه، بررسی تأثیر مکمل یاری ویتامین D مقاومت به انسولین در مبتلایان به دیابت است. روش بررسی: نوع مطالعه مرور سیستماتیک و متا‌آنالیز می‌باشد که در واقع یک مطالعه مروری روی بررسی‌های انجام شده موجود است. ابتدا جستجوی وسیعی بر روی منابع الکترونیکی انگلیسی شامل EMBASE, Web of science, Medline Scopus و Who و نیز منابع الکترونیکی فارسی شامل SID و Iranpsych برای یافتن مقالات پژوهشی مرتبط با دیابت انجام شد. یافته‌ها: با توجه به آزمون ناهمگنی، مطالعات دارای ناهمگنی معنی‌داری در ویتامین D، HOMA-IR و حساسیت انسولین بودند. بنابراین از مدل اثرات تصادفی استفاده شد و اختلاف میانگین و فاصله اطمینان 95% بین دو گروه مورد و شاهد به ترتیب در ویتامینD، 87/1 و (23/9،49/5-)، همچنین درHOMA-IR، 09/0 و (05/0،23/0-) و حساسیت انسولین، 24/0 و (26/0،75/0-) بود. نتیجه‌گیری: به طور‌کلی نتایج مطالعه حاضر نشان داد که با توجه به متا‌آنالیز، تجویز ویتامین D و میزان حساسیت انسولین و HOMA-IR بر روی افراد دیابتی تأثیرگذار نمی‌باشد

Title: بررسی سبک زندگی سالم کارکنان مراکز بهداشتی درمانی اندیمشک

http://tbj.ssu.ac.ir/article-1-1901-fa.html

چکیده مقدمه: کارکنان بخش سلامت نقش مهمی را در آموزش سبک زندگی سالم به گروههای مختلف جامعه بر عهده دارند و به عنوان الگوی مناسب رعایت سبک زندگی سالم به حساب می‌آیند. بنابراین این پژوهش با هدف بررسی سبک زندگی سالم کارکنان مراکز بهداشتی درمانی اندیمشک انجام گرفت. روش‌بررسی: در این پژوهش توصیفی– تحلیلی کلیه کارکنان بهداشتی درمانی(170 نفر) به روش سرشماری انتخاب شدند. برای جمع‌آوری اطلاعات از پرسشنامه استاندارد رفتارهای ارتقا‌دهنده سلامت (HPLPII: Health promotion lifestyle profile II) استفاده شد. تجزیه و تحلیل داده‌ها توسط نرم‌افزارspss نسخه 19و آزمون‌های آماری توصیفی و آزمون تی‌مستقل، آنالیز واریانس یک‌طرفه و همچنین ضریب همبستگی Pearson انجام شد. یافته‌ها: میانگین سنی آنها 06/7±25/36 سال بود. 8/26 درصد مرد و 1/77 درصد، متاهل بودند. نمره کل سبک زندگی در کارکنان 34/15±130 بدست آمد. بیشترین امتیاز را بعد مسئولیت‌پذیری درباره سلامت (67/4±90/28) و کمترین امتیاز را فعالیت فیزیکی (67/4±90/28)کسب کرد. بین متغیرهای دموگرافیک با سبک زندگی کلی ارتباط آماری معنی‌ دار وجود نداشت(05/0p<). ولی بین جنس و تغذیه(005/0=P)، تاهل و روابط بین فردی (005/0=P)، BMI و روابط بین فردی(005/0=P)، سابقه کار و رشد معنوی(016/0=P) ارتباط آماری معنی‌دار مشاهده شد. نتیجه‌گیری: یافته‌های این پژوهش نشان دهنده این است که انجام رفتارهای ارتقا‌دهنده سلامت در کارکنان بهداشتی از سطحی متوسط برخوردار می‌باشد. بنابراین برنامه‌ریزی جهت بهبود و ارتقای سبک زندگی این گروه شغلی پر اهمیت که نقش بسیار مهمی در ارتقای سلامتی جامعه دارند ضروری به نظر می‌رسد.

Title: مطالعه سینتیک واکنش و ایزوترم های جذب رنگ متیلن بلو بوسیله پوکه معدنی طبیعی از محیط‌های آبی

http://tbj.ssu.ac.ir/article-1-1902-fa.html

مقدمه: گروه رنگ‌زای آزو به‌طور گسترده برای رنگ رزی الیاف مورد استفاده قرار می‌گیرند. متیلن بلو یکی از رنگ‌های این گروه می‌باشد که بدلیل وجود حلقه‌های بنزنی در ساختار آن موجب سمی بودن و ماندگاری آن در محیط می‌شود. عدم تصفیه مناسب آن، صدمات جبران‌ناپذیری به محیط زیست وارد می‌کند. یکی از راه‌های حذف این رنگ از منابع طبیعی جذب آن بوسیله جاذب‌ها می‌باشد. لذا هدف از این مطالعه بررسی سینتیک واکنش و ایزوترم های جذب رنگ متیلن بلو پامیس پودری می‌باشد. روش بررسی: فرایند جذب در شرایط آزمایشگاهی در pH خنثی،غلظت ثابت رنگ و دوزهای مختلف جاذب مورد بررسی قرار گرفت. سپس غلظت باقیمانده در محلول رنگ از طریق جذب بوسیله با دستگاه اسپکتروفتومتر (Cary 50 ساخت کمپانی (Perkin Elmerدر طول موج nm 665اندازه‌گیری شد. به منظور درک چگونگی جذب، داده های بدست آمده با ایزوترم‌های جذب لانگمیر، فروندلیچ، تمکین، هارکینز- جورا، دوبینین-رادوشکویچ و سینتیک های واکنش شبه درجه اول، شبه درجه دوم، پخش درون ذره‌ای و الوویچ برازش شدند. یافته‌ها: نتایج نشان داد در بین پنج مدل ایزوترمی مورد بررسی فرایند جذب از ایزوترم هارکینز- جورابا 9914/0R2= بیشترین تبعیت را دارد، همچنین چگونگی جذب از هر دو ایزوترم لانگمیر و فروندلیچ نیز تبعیت مناسبی دارد‌(9633/0 R2= 9365/0R2=) و این بدین معنی است که فرایند جذب هم به صورت تک لایه‌ای و هم چند‌لایه‌ای صورت می‌گیرد. پایین بودن متوسط انرژی آزاد(KJ/mol323/0E=) در مدل دوبینین- رادوشکویچ نشان داد جذب فیزیکی فرایند غالب جذب است. همچنین فرایند جذب از واکنش شبه درجه دوم (9998/0R2=) تبعیت می‌کند. نتیجه‌گیری: با توجه به نتایج می‌توان گفت جذب رنگ متیلن‌بلو به صورت چند‌لایه‌ای بیشتر از تک‌لایه‌ای صورت می‌گیرد و پوکه معدنی جاذبی کارا برای حذف رنگ متیلن بلو می‌باشد(2/0RL=).

Title: بررسی وضعیت امنیت غذایی سالمندان و عوامل مرتبط با آن در شهر اراک در سال 1391

http://tbj.ssu.ac.ir/article-1-1904-fa.html

چکیده مقدمه: امروزه افزایش امید به زندگی، کاهش میزان مرگ و میر و بهبود شرایط بهداشتی موجب بالا رفتن شمار سالمندان، به عنوان یکی از گروههای آسیب پذیر جامعه شده است. بنابراین امنیت غذایی یکی از پیش نیازهای ضروری برای سلامت سالمندان است. مطالعه حاضر به منظور بررسی وضعیت امنیت غذایی سالمندان وعوامل مرتبط با آن طراحی واجرا شده‌است. روش بررسی: این مطالعه توصیفی مقطعی درسال 1391 و با مشارکت 300 نفر از سالمندان شهر اراک انجام شده است. روش بررسی، با استفاده از پرسش نامه امنیت غذایی FaCPS-FSSM اعتبارسنجی شده برای سالمندان با انجام مصاحبه صورت گرفت. تجزیه و تحلیل داده‌ها با استفاده از نرم افزار SPSS و بکارگیری آزمون آمای کای اسکویر، t مستقل، ضریب همبستگی پیرسون و رگرسیون انجام پذیرفت. یافته‌ها: نتایج نشان داد که 3/39 درصد از سالمندان دارای امنیت غذایی کامل، 29 درصد ناامن غذایی بدون گرسنگی، 7/20 درصد ناامنی غذایی متوسط و 11 درصد ناامنی غذایی شدید میباشند. ارتباط معنی‌داری بین سطح سواد، شغل، وضعیت تاهل، نمایه توده بدن، ابتلا به بیماری‌های مختلف و بعد خانوار با امنیت غذایی حاصل شد(05/0>p). نتیجه‌گیری: با توجه به اینکه نزدیک به 7/60 درصد از سالمندان مورد مطالعه از درجاتی از ناامنی غذایی به سر می‌بردند، همچنین با توجه به عوامل مختلفی که در این مطالعه رابطه معنی‌داری با این مشکل داشتند، لزوم توجه به این مشکل در این طبقه از جمعیت آسیب‌پذیر جامعه بیش از پیش ضرورت پیدا کرده است.

Title: رابطه عدالت سازمانی و رفتار شهروندی سازمانی

http://tbj.ssu.ac.ir/article-1-1905-fa.html

مقدمه: اگر مدیران خواهان کارکنانی هستند که به فراسوی انتظارات می روند، به میل و خواست خود به رفتارهایی دست می‌زنند که جزء وظایف رسمی شغلی‌شان نیست، و به طور کلی رفتارشهروندی سازمانی بالایی دارند باید با کارکنان در محیط کاری رفتاری عادلانه داشته باشند. در این راستا، مطالعه حاضر به دنبال بررسی رابطه عدالت سازمانی با رفتار شهروندی سازمانی در کارکنان مرکز آموزشی، درمانی و تحقیقاتی روان پزشکی بهاران زاهدان می‌باشد. روش بررسی: مطالعه حاضر از نوع توصیفی- همبستگی بوده که به شیوه نمونه‌گیری طبقه‌ای- تصادفی تعداد 65 پرسشنامه بین کارکنان مرکز آموزشی، درمانی و تحقیقاتی روان پزشکی بهاران زاهدان توزیع شد. ابزار جمع‌آوری اطلاعات در این مطالعه 2 پرسشنامه معتبر عدالت سازمانی نی هوف و مورمن (1993) و رفتار شهروندی سازمانی پودساکف و همکاران (1990) بود. برای تجزیه و تحلیل داده‌ها از آزمون ضریب همبستگی اسپیرمن و رگرسیون چندگانه با استفاده از نرم افزار SPSSنسخه 17 استفاده شد. یافته‌ها: 1- ادراک کارکنان از وضعیت عدالت سازمانی نسبتا مطلوب می‌باشد. 2- کارکنان مورد مطالعه رفتار شهروندی سازمانی نسبتا مطلوبی را از خود بروز می‌دهند.3- بین عدالت سازمانی و ابعاد سه گانه آن با رفتار شهروندی سازمانی رابطه مثبت معنی‌داری وجود دارد.4- نتایج رگرسیون چندگانه نیز نشان داد از بین ابعاد سه‌گانه عدالت سازمانی، دو بعد عدالت توزیعی و تعاملی قابلیت پیش‌بینی رفتار شهروندی را دارند. نتیجه‌گیری: با توجه به محرز شدن نقش مثبت عدالت سازمانی در بروز رفتار شهروندی سازمانی برگزاری دوره‌های آموزشی جهت آشنایی مدیران سازمان‌ها با مبانی و اصول عدالت سازمانی، تعاملات انسانی و نحوه به کارگیری آنها در سازمان‌ها پیشنهاد می‌گردد.

Title: پیش‌تغلیظ و اندازه‌گیری آنتیموان در آب آشامیدنی بطری شده با استفاده از نانوذرات آلومینیم اکسید اصلاح شده

http://tbj.ssu.ac.ir/article-1-1906-fa.html

چکیده مقدمه: آنتیموان تری اکسید (Sb2O3) به عنوان یک کاتالیزور در تولید پلی اتیلن ترفتالات(PET)  استفاده می‌شود و مطالعات انجام شده روی آب‌های بطری‌شده نشان می‌دهد که آنتیموان می‌تواند از بطری‌های پلاستیکی پلی اتیلن ترفتالات(PET)  به داخل آب آشامیدنی نشت کند. روش بررسی: در این مطالعه یک روش ساده برای تعیین مقادیر کمی آنتیموان در آب آشامیدنی بطری شده استفاده شده است. که بر اساس پیش تغلیظ/ استخراج فاز جامد است. نانو ذره آلومینیوم اکسید اصلاح شده با یک لیگاند شیف باز برای پیش تغلیظ استفاده شد. آزمایش‌ها در یک سیستم مداوم انجام شد و ازHCl  به عنوان شوینده استفاده شد. پارامترهای شیمیایی و حجمی برای پیش‌تغلیظ و اندازه‌گیری آنتیموان بهینه شدند. غلظت آنتیموان توسط طیف‌سنجی جذب اتمی اندازه‌گیری شد.  برای بررسی اثر عوامل محیطی در میزان ورود آنتیموان در آب (PET)، بطری‌ها در شرایط مختلف‌(دمای اتاق، در معرض نور خورشید و ºC 18-) نگهداری شدند. یافته‌ها: نمودار کالیبراسیون در محدوده ppm 15-50/0 آنتیموان، با حد تشخیص ppm 055/0 به صورت خطی رسم شد. مقدار بهینه حجم نمونهmL  450، سرعت عبور نمونه از جاذبmL/min  5، حجم شویندهmL  5 و سرعت عبور شوینده از جاذبmL/min  2 بدست آمد. نتیجه‌گیری: نتایج نشان داد که نانوآلومینای اصلاح شده یک جاذب موثر در جدب آنتیوان از آب وHCL 1M  محلول شوینده مناسب می‌باشد. ماکزیمم آزاد شدن آنتیموان از آب بطری شده وقتی بطری آب در معرض نور خورشید قرار می‌گیرد انجام می‌شود.

Title: بررسی تاثیرآموزش بررفتار خودمراقبتی و منافع و موانع انجام آن دربیماران تحت درمان همودیالیز در سال 1392

http://tbj.ssu.ac.ir/article-1-1907-fa.html

مقدمه: نارسایی مزمن کلیه یکی از شایع‌ترین بیماری مردم درتمام کشورهای دنیاست. خودمراقبتی یکی از جنبه‌های مهم درمان در بیماران همودیالیزی محسوب می‌شود ولازم است که آموزش خودمراقبتی در برنامه‌های درمانی این بیماران در بیمارستان‌ها قرار گیرد. این مطالعه با هدف تعیین بررسی تاثیرآموزش بر رفتار خودمراقبتی و منافع و موانع انجام آن دربیماران همودیالیز انجام شد. روش بررسی: در این مطالعه تجربی، تعداد 40 نفر مورد و40نفر شاهد به روش نمونه‌گیری تصادفی از بین بیماران تحت درمان در بخش همودیالیز بیمارستان امام رضا(ع) آمل و امام خمینی(ره) نور‌ وابسته به دانشگاه علوم پزشکی مازندران، درسال 1392 انتخاب شدند. بیماران گروه آزمون در طی سه جلسه25-30 دقیقه‌ای، تحت آموزش قرار گرفتند. داده‌ها از طریق پرسشنامه و طی مصاحبه با بیماران، قبل و بعد از مداخله، جمع‌آوری شد و با استفاده از نرم افزار 18 SPSS و آزمون‌های   Anova , t-test، ضریب همبستگی و تحلیل رگرسیون، تجزیه و تحلیل گردید. یافته‌ها: پس ازمداخله آموزشی نمره‌‌ رفتارهای خودمراقبتی همودیالیز و منافع انجام آن‌ها بطور معنی‌داری نسبت به قبل ازآموزش‌ در گروه آزمون افزایش وموانع انجام آن کاهش معنی‌دار داشته، در حالی که گروه شاهد هیچ تغییر قابل‌توجهی در این متغییرها نداشت. نتیجه‌گیری: برنامه آموزشی اجراشده در این مطالعه در بهبود رفتارهای خودمراقبتی این بیماران و مثبت نمودن درک آنها از منافع و موانع انجام آن موثر بوده است. پزشکان و پرستاران بخش دیالیز بیمارستان‌ها با بکارگیری این روش آموزشی، می‌توانند نقش بسیار موثرتری در بهبود رفتارهای خودمراقبتی این بیماران و ارتقاءسلامت بیماران همودیالیزی و بالطبع افزایش کیفیت زندگی و طول عمر آنها داشته باشند.

Title: بررسی نگرش و رفتارهای خودمراقبتی بیماران مبتلا به استئوآرتریت زانو مراجعه‌کننده به مراکز کلینیک روماتولوژی شهرستان یزد در سال 1391

http://tbj.ssu.ac.ir/article-1-1908-fa.html

چکیده مقدمه: استئوآرتریت زانو از شایعترین علل درد مفصل زانو وابسته به سن می‌باشد که باعث ناتوانی، از کار افتادگی و کاهش کیفیت زندگی می‌شود. هدف پژوهش حاضر بررسی سطح نگرش و رفتارهای خودمراقبتی بیماران استئوآرتریت زانو مراجعه کننده به سه کلینیک روماتولوژی شهرستان یزد می‌باشد. روش‌بررسی: این پژوهش توصیفی- تحلیلی بر روی 235 بیمار مراجعه‌کننده به مراکز درمانی شهرستان یزد که بصورت تصادفی و داوطلبانه انتخاب شدند، انجام شد. ابزار گردآوری اطلاعات پرسشنامه شامل متغیرهای دموگرافیک، سوالات نگرش و رفتارهای خودمراقبتی بود که روایی و پایایی پرسشنامه مورد تایید قرارگرفت. داده‌ها با استفاده از نرم‌افزار SPSS نسخه 18 و با آزمون‌های آماری پارامتریک t-test، ANOVA، ضریب همبستگی، 2&chi; در سطح معنی‌داری 05/0 مورد تجزیه و تحلیل قرار گرفتند. یافته‌ها: میانگین سنی افراد مورد مطالعه 15/9±9/54 سال و BMI آنها 6/4±8/28 بود. میانگین نمره نگرش بیماران نسبت به خودمراقبتی 95/3±/41/47 از 55 نمره و نمره خودمراقبتی بیماران 75/5±11/43 از 60 نمره بود که هر دو امتیاز در سطح متوسط می‌باشد. همچنین رابطه آماری معنی‌دار مثبتی بین نگرش و رفتارهای خودمراقبتی مشاهده گردید(01/0P=). در بین رفتارهای مختلف خودمراقبتی، نگرش بیماران به تاثیر مثبت استفاده از عصا در خودمراقبتی بیماری در کمترین میزان بود و مطابق با اعتقاد بیماران، در بین رفتارهای خودمراقبتی استفاده ازعصا در حین راه رفتن کمترین عملکرد را دارا بود. نتیجه‌گیری: با توجه به نتایج مبنی برمتوسط بودن میزان نگرش و رفتارهای خودمراقبتی بیماران و نیز همبستگی مثبت بین آنها با بهبود نگرش، خودمراقبتی بیماران نیز افزایش می‌یابد؛ بعلاوه با توجه به این که در بین رفتارهای خودمراقبتی نگرش استفاده از عصا، استخر رفتن و کاهش وزن در سطح پایین‌تر بود‌، بنابراین توصیه می‌شود در برنامه‌های مداخله‌ای برموارد مذکور تاکید بیشتری شود. مقاله حاضر برگرفته از پایان نامه تحصیلی مقطع کارشناسی ارشد آموزش بهداشت دانشکده بهداشت دانشگاه علوم پزشکی و خدمات بهداشتی درمانی شهید صدوقی یزد می باشد.

Title: بررسی کیفیت زندگی بیماران عضو انجمن MS شهر یزد

http://tbj.ssu.ac.ir/article-1-1909-fa.html

مقدمه: بیماری ام اس یک بیماری مزمن و پیش رونده سیستم اعصاب مرکزی است که دارای علایم متعدد می باشد.این نشانه ها اثرات منفی زیادی روی کیفیت زندگی بیماران می گذارد.این مطالعه با هدف ارزیابی کیفیت زندگی بیماران عضو انجمن ام اس در شهر یزد طراحی و اجرا گردید. روش بررسی:پژوهش حاضر توصیفی- مقطعی است.جامعه آماری شامل بیماران عضو انجمن ام اس شهر یزد بودند.تعداد افراد نمونه 108 نفر بود که به صورت تصادفی(مراجعه تصادفی پژوهشگر در روزهای مختلف به انجمن در فاصله زمانی شهریور تا پایان مهر ماه 1392 انتخاب و وارد مطالعه شدند و اطلاعات دموگرافیک آنان ثبت شد.روش گرد آوری داده ها به صورت خود گزارشی  وبا استفاده از پرسشنامه اختصاصی کیفیت زندگی بیماران مولتیپل اسکلروزیس)54-MSQOL)بود که حاوی54 سوال می باشد.د اده ها توسط نرم افزار18-SPSS وآمار توصیفی و آزمونهای آماری T-TEST ،همبستگی پیرسون و اسپیرمن تحلیل گردید. یافته ها: میانگین سنی شرکت کنندگان در این مطالعه 31/9±27/34 سال بود. بالاترین میزان نمره در بین14خرده مقیاس کیفیت زندگی مربوط به عملکرد اجتماعی(57/25±45/61) و کمترین میزان مربوط به محدودیت نقش به علت مشکلات جسمی (64/37±37/34)  می باشد. میانگین نمرات حیطه ترکیبی سلامت جسمانی در افراد  نمونه(87/12±29/49) و سلامت روانی (62/20±38/38) بدست آمد.  بین دو جنس از نظر حیطه های ترکیبی کیفیت زندگی(ابعاد سلامت جسمانی و روانی)، تفاوت معناداری وجود ندارد(05/0&le;P ). بین  ابعاد جسمانی و روانی کیفیت زندگی  و همچنین هریک از این دو بعد  با تحصیلات رابطه مستقیم و معنادار وجود داشت (05/0&ge;p ). بر اساس نتایج تحلیل همبستگی پیرسون، بین سن و مدت زمان ابتلا به بیماری  با ابعاد دوگانه کیفیت زندگی رابطه معکوس و معنادار وجود داشت (05/0&ge;p ). نتیجه گیری: در این مطالعه کیفیت زندگی بیماران عضو انجمن ام اس شهرستان یزد کمتر از حد متوسط می باشد.بیماری ام اس بر تمامی ابعاد زندگی فرد اثر منفی دارد.از این رو آنچه در این زمینه حایز اهمیت است، طراحی خدماتی همه جانبه جهت ارتقای کیفیت زندگی این گروه از بیماران می باشد.

Title: بررسی پایداری، سمیت و اثر ضد میکروبی محلول آلیسین

http://tbj.ssu.ac.ir/article-1-1911-fa.html

چکیده مقدمه: آلیسین از عصاره سیر گرفته شده و به واسطه گروه تیوسولفونات توانایی اتصال به گروه‌های تیول پروتئین‌ها را دارد. این اتصال باعث تخریب انواع پروتئین‌ها و آنزیم‌های مهم میکروب‌ها شده و می‌تواند بر طیف وسیعی از ویروس‌ها، باکتری‌ها، قارچ‌ها و انگل‌ها موثر باشد. هدف این مطالعه بررسی پایداری، سمیت و اثر ضد میکروبی آلیسین محلول می‌باشد. روش بررسی: نخست سریال غلظت (31،62، 125، 250، 500 میکروگرم در میلی‌لیتر) محلول آلیسین تهیه و در معرض سوسپانسیون سوش‌های استاندارد باکتریایی شامل سودوموناس آئروژینوزا(ATTC 25987)، اشریشیاکلی(ATTC 25922) و استافیلوکوکوس اوررئوس(ATTC 25923) و سوش‌های استاندارد قارچی شامل کاندیدا آلبیکنس(ATTC 10231) و آسپرژیلوس نیجر(ATTC 16888) قرار داده شد و در نهایت کمترین غلظت مهاری(MIC50 وMIC90) این ترکیب علیه هر سوش، تعیین گردید. برای بررسی سمیت، نخست سوسپانسیون سلول‌های پوست موش، نژاد بالب سی تهیه و به مدت 6، 12 و 24 ساعت در معرض سریال غلظت محلول آلیسین قرار گرفت. سپس با تست MTT میزان حیات سلولی با توجه به کنترل محاسبه گردید. برای بررسی پایداری محلول آلیسین، نخست تعدادی قطعه سنگ مرمر استریل شده تهیه و به طور جداگانه روی سطح آنها با محلول آلیسین آغشته گردید. بعد از گذشت  6، 12 و 24 ساعت از قطعه سنگ‌ها نمونه‌گیری و روی پلیت نوترینت آگار تلقیح شد. محیط‌های کشت به مدت 48 ساعت در 37 درجه سانتی‌گراد انکوبه شده و در نهایت تعداد کلونی‌های رشد کرده در هر پلیت، شمارش گردید. یافته‌ها: تست میکرودایلوشن نشان داد کمترین میزان MIC50 برای محلول آلیسین مقدار 62 میکروگرم بر میلی لیتربرای سوش کاندیدا و بیشترین میزان MIC50 برای سوش استافیلوکوکوس اورئوس مشاهده گردید. محلول آلیسین بر روی همه سوش‌های باکتریایی و قارچی مورد مطالعه اثر ضد میکروبی دارد. همچنین این مطالعه به ما نشان داد که سمیت محلول آلیسین به مقدار اندکی وابسته به زمان و غلظت بوده ولی افزایش زمان تا 24 ساعت تاثیر چشمگیری بر کاهش پایداری این ماده نداشت. نتیجه‌گیری: بر اساس نتایج محلول آلیسین دارای خاصیت ضدمیکروبی بوده و سمیت آن ناچیز و قابل اغماض می‌باشد. همچنین این ماده از پایداری بالایی در شرایط محیطی برخوردار است.

Title: ارزیابی روشنایی در کارگاه‌های بافندگی شهر یزد

http://tbj.ssu.ac.ir/article-1-1910-fa.html

مقدمه: روشنایی خوب، باعث بالا رفتن بازده کار و همچنین اثرات مفید بر حالات فیزیولوژیک عمومی افراد دارد. رابطه معنی‌داری بین روشنایی کم و فراوانی بالای حوادث ناشی از کار وجود دارد. با توجه به اهمیت روشنایی و نظر به اینکه کارگاه‌های بافندگی به دلیل کار دقیق و حساس و عدم نظارت ارگان‌های مربوطه به روشنایی بالایی نیاز دارند، این مطالعه با هدف ارزیابی روشنایی در کارگاه‌های بافندگی شهر یزد در دو فصل بهار و پاییز و بهبود روشنایی در کارگاه‌های با شرایط روشنایی ضعیف صورت گرفت. روش‌بررسی: این پژوهش، در10 کارگاه خانگی بافندگی شهر یزد انجام شد. اندازه‌گیری‌ها در خردادماه به دلیل وجود حداکثر روشنایی طبیعی و آبان‌ماه در روزهای ابری سال صورت پذیرفت. بنابر‌این در هر دو فصل سال روشنایی کلی، طبیعی و مصنوعی مورد بررسی قرار گرفت. بدین صورت که به منظور اندازه‌گیری نور طبیعی تمام منابع روشنایی مصنوعی(لامپ)خاموش گردید و پس از اندازه‌گیری، با روشن نمودن مجدد لامپ‌ها، میزان روشنایی کلی (طبیعی و مصنوعی) اندازه‌گیری شد. به منظور اندازه‌گیری روشنایی عمومی از الگوی ارایه شده توسط Illuminating Engineering society و ایستگاه سطوح کار استفاده گردید. با توجه به اینکه اپراتور اکثر ساعات کاری را در قسمت چله و جلوی دستگاه سومت سپری میکرد اندازه‌گیری‌های موضعی در این دو قسمت صورت گرفت. در پایان کارگاه‌های با روشنایی کمتر از حد استاندارد شناسایی و مداخلاتی جهت بهبود شرایط موجود انجام گرفت و مجددا روشنایی این کارگاه‌ها اندازه‌گیری شد.    یافته‌ها: حداقل و حداکثر میانگین شدت روشنایی کلی در فصل بهار به ترتیب مربوط به کارگاه‌های شماره 4 و 6 با مقادیر 67/278 و 53/452 لوکس می‌باشد.کارگاه‌های شماره 5 و 2 با مقادیر 47/184 و 43/218 لوکس به ترتیب دارای حداقل و حداکثر مقدار نور روز در فصل بهارمی‌باشند. کمترین و بیشترین میانگین شدت روشنایی کلی در فصل پاییز به ترتیب مربوط به کارگاه‌های 2 و 6 با مقادیر 13/230 و 57/400 لوکس و همچنین حداقل و حداکثر میانگین شدت روشنایی طبیعی به ترتیب مربوط به کارگاه‌های 6 و 3 با مقادیر 37/151 و 77/183 لوکس می‌باشد. با انجام طراحی مجدد و یک سری مداخلات نظیر: تعویض لامپ‌ها، تمیز کردن سطوح و لامپ‌ها روشنایی کارگاه شماره 4،2 به میزان حد استاندارد رسید. نتیجه‌گیری: اکثر کارگاه‌های بافندگی مورد مطالعه دارای روشنایی مطلوبی بوده که البته با تدابیری همچون چیدمان صحیح و نظافت و گردگیری دوره‌ای لامپ‌ها، تعویض مرتب چراغ‌های سوخته و استفاده از لامپ فلورسنت می‌توان شرایط روشنایی محیط را بهبود بخشید.

Title: بررسی رابطه جهت گیری مذهبی و گرایش به تفکر انتقادی با راهبردهای مقابله با استرس در دانشجویان دختر دوره کارشناسی دانشکده علوم انسانی دانشگاه یزد

http://tbj.ssu.ac.ir/article-1-1912-fa.html

چکیده مقدمه: برخورداری از باورهای دینی و نیروی تفکر نقش مهمی در کاهش اختلالات روانی و استرس دارد. لذا پژوهش حاضر با هدف بررسی رابطه بین جهت گیری مذهبی و گرایش به تفکر انتقادی با راهبردهای مقابله با استرس در دانشجویان دختر دوره کارشناسی دانشکده علوم انسانی دانشگاه یزد انجام گرفت. روش بررسی: جامعه­ی آماری پژوهش شامل 1617 دانشجوی دختر دوره کارشناسی دانشگاه یزد بود که از این جامعه بر اساس جدول مورگان-کرجسی تعداد 300 نفر از دانشکده­ی علوم انسانی به عنوان نمونه به روش تصادفی طبقه­ای با نسبت مساوی انتخاب شدند. برای سنجش متغیّرهای پژوهش از پرسشنامه­ی جهت گیری مذهبی آلپورت، پرسشنامه­ی گرایش به تفکر انتقادی ریکتس و پرسش نامه مقابله با موقعیت های فشارزای اندلر و پارکراستفاده شد. تحلیل داده­ها نیز با استفاده از ضریب همبستگی پیرسون و تحلیل رگرسیون چند گانه صورت گرفت. یافته­ها: ابتدا بین جهت گیری مذهبی درونی و سپس جهت گیری بیرونی با سبک مقابله مساله مدار و همچنین بین گرایش به تفکر انتقادی و سبک مساله مدار هم بستگی مثبتی در دانشجویان مشاهده شد. از بین مولفه های گرایش به تفکر انتقادی، مولفه نوآوریی و بلوغ فکری با سبک مساله مدار و اشتغال ذهنی در ابتدا با سبک مساله مدار و سپس با سبک اجتنابی هم بستگی مثبت معنادار و در مقابل، مولفه بلوغ فکری نیز با دو سبک هیجان مدار و اجتنابی همبستگی منفی معناداری داشتند. نتیجه­گیری: با توجه به یافته‌های پژوهشی با آموزش تفکر انتقادی و تلاش در جهت درونی سازی مذهب می‌توان بهره‌گیری دانشجویان از سبک‌های مقابله‌ای کارآمد در هنگام مواجهه با  رویدادهای تنش‌زا را افزایش داد.

Title: اثرجریان الکتریسته مستقیم بر حیات انگل سالک شهری درمحیط کشت

http://tbj.ssu.ac.ir/article-1-1913-fa.html

مقدمه: لیشمانیوز که جزء ده بیماری مهم عفونی دنیاست از جمله سه بیماری مهم است که توسعه در زمینه واکسن ،دارو و حشره کش برای کنترل آن با مشکل مواجه هست. تحریک الکتریکی به عنوان روش درمانی در تعدادی از بیماریها مورد استفاده قرار گرفته است. اما برروی درمان لیشمانیوز جلدی به طور معمول به کار نرفته است. لذا هدف ما در این بررسی اثرجریان الکتریسته مستقیم بر حیات انگل  سالک شهری درمحیط کشت می باشد.    روش بررسی: مطالعه از نوع تجربی و به شیوه کارآزمایی آزمایشگاهی بود. انگل لیشمانیا گونه سالک شهری ایران کشت داده شدو پروماستیگوت های هردو مرحله پویا و ایستا تهیه شد. الکترود ها در فاصله 2 یا 4 سانتیمتری از هم بود و وقتی ولتاژ به ترتیب 5/2، 5/3 و 5/4 به الکترودها اعمال شده بود در زمانهای 5، 10، 15، 20، 25، 30، 35 و 70 دقیقه تعداد پروماستیگوت های زنده نمونه شمارش شد. داده ها ثبت و مورد تجزیه و تحلیل قرار گرفت. یافته ها :  میانگین پروماستیگوت های زنده مرحله ایستا و پویا وقتی که فاصله الکترودها 2 سانتی متر بود برای ولتاژ های 5/2، 5/3 و 5/4 در مقایسه باگروه شاهد اختلاف معنی دار نشان داد (003/0 =p ).  همچنین  این تفاوت برای ولتاژ 5/2 و 5/4 ولت  هم معنی دارشد(004/0 =p ). ولی پروماستیگوت های زنده مرحله پویا برای ولتاژ 5/3 و 5/4 ولت معنی دار نشد (05./ =p ). پروماستیگوت های زنده مرحله ایستا وقتی که فاصله الکترودها 4 سانتی متر بود برای ولتاژ 5/2 و5/3 ولت معنی دار نشد (148/ 0=p ). ولی این تفاوت برای ولتاژ 5/2 و 5/4 ولت  معنی دار شد(0001/0 =p ). ولی برای ولتاژ 5/3 و 5/4 ولت هم معنی دار شد 001/0/p<)، پروماستیگوت های زنده مرحله پویا وقتی که فاصله الکترودها 4 سانتی متر بود، تفاوت میانگین تعداد انگلها برای ولتاژ 5/2 و5/3 ولت معنی دارنشد (725/0 =p ).  این تفاوت برای ولتاژ 5/2 و 5/4 ولت  معنی دار (004/0 =p ). و همچنین برای  ولتاژهای 5/3 و 5/4 ولت نیز معنی دار شد (007/0 =p ). نتیجه گیری: انگل به جریان مستقیم الکتریسیته در محیط کشت حسای و پروماستیگوت های پویا حساس تر و هرچه فاصله الکترودها کمتر و ولتاژ بیشتر باشد تاثیر جریان الکتریسیته بر کنترل رشد بیشتر هست.
